# Disorder of neuroplasticity aggravates cognitive impairment via neuroinflammation associated with intestinal flora dysbiosis in chronic heart failure

**DOI:** 10.18632/aging.205960

**Published:** 2024-07-01

**Authors:** Jie Chen, Xiaohong Wei, Xuefen Wu, Qian Zhang, Guiyang Xia, Huan Xia, Hongcai Shang, Sheng Lin

**Affiliations:** 1 Key Laboratory of Chinese Internal Medicine of Ministry of Education and Beijing, Dongzhimen Hospital, Beijing University of Chinese Medicine, Beijing 100700, China

**Keywords:** cognitive impairment, chronic heart failure, synaptic plasticity, neuroinflammation, intestinal flora dysbiosis

## Abstract

Background: Chronic heart failure (CHF) impairs cognitive function, yet its effects on brain structure and underlying mechanisms remain elusive. This study aims to explore the mechanisms behind cognitive impairment.

Methods: CHF models in rats were induced by ligation of the left anterior descending coronary artery. Cardiac function was analyzed by cardiac ultrasound and hemodynamics. ELISA, immunofluorescence, Western blot, Golgi staining and transmission electron microscopy were performed on hippocampal tissues. The alterations of intestinal flora under the morbid state were investigated via 16S rRNA sequencing. The connection between neuroinflammation and synapses is confirmed by a co-culture system of BV2 microglia and HT22 cells *in vitro.*

Results: CHF rats exhibited deteriorated cognitive behaviors. CHF induced neuronal structural disruption, loss of Nissl bodies, and synaptic damage, exhibiting alterations in multiple parameters. CHF rats showed increased hippocampal levels of inflammatory cytokines and activated microglia and astrocytes. Furthermore, the study highlights dysregulated PDE4-dependent cAMP signaling and intestinal flora dysbiosis, closely associated with neuroinflammation, and altered synaptic proteins. *In vitro*, microglial neuroinflammation impaired synaptic plasticity *via* PDE4-dependent cAMP signaling.

Conclusions: Neuroinflammation worsens CHF-related cognitive impairment through neuroplasticity disorder, tied to intestinal flora dysbiosis. PDE4 emerges as a potential therapeutic target. These findings provide insightful perspectives on the heart-gut-brain axis.

## INTRODUCTION

The heart and brain exhibit pathophysiological crosstalk. This relationship is markedly shaped by the cardio-brain axis, which describes the impact of heart activities on cerebral functions like language, emotion, and memory [1]. The idea that the heart governs mental activities dates back two thousand years to the ancient Chinese text, Huangdi Neijing. Chronic heart failure (CHF) remains a significant contributor to morbidity and mortality, threatening approximately 1%–2% of adults, with a growing incidence among younger individuals worldwide [2]. A considerable proportion of CHF patients, ranging from 25%–75%, experience a decline in cognitive functions, including memory deterioration, attention deficit, and depression [3, 4]. Notably, CHF patients with cognitive impairment at discharge face a 5-fold increased risk of all-cause readmission or death as a composite endpoint and a 4-fold increased risk of readmission [5]. Given the high prevalence and severe outcome of post-CHF cognitive impairment, it represents a significant public health concern. The gradual recognition of cognitive impairment as a determinant of adherence to CHF treatment necessitates its incorporation into the individualized management of CHF patients [6]. Despite this, research on cognitive impairment following CHF remains limited. As such, it is imperative to investigate the mechanisms underlying the vicious circle of cognitive impairment and CHF, and to develop novel therapeutic approaches to address them.

The gut, sharing numerous neurological functions with the brain and impacting emotional and mental states, is often designated as the “second brain” [7]. Recent findings indicate that intestinal microbiota dysbiosis is linked to immune dysfunction in the brain, resulting in cognitive decline [8, 9]. A recent investigation validated the presence of intestinal flora dysbiosis following CHF, which resulted in elevated levels of inflammatory cytokines [10]. The notion that neuroinflammation, characterized predominantly by activated microglia and astrocytes, is a pivotal pathological hallmark of mild cognitive impairment and a central pathogenic mechanism, is supported by mounting evidence [11–13]. The activation of microglia and astrocytes may trigger the release of pro-inflammatory cytokines, such as tumor necrosis factor-α (TNF-α) and interleukin-1β (IL-1β), potentially causing neuronal death, hindering long-term potentiation (LTP) and hippocampal neurogenesis, and compromising synaptic function and integrity in critical cerebral regions, including the cortex, striatum, and hippocampus [14–17].

Neuronal hyperactivity can be mediated by synaptic mechanisms, such as increased excitatory neurotransmission or decreased inhibitory neurotransmission [18]. Synaptic plasticity, a crucial aspect of synaptic function, pertains to the activity-dependent modifications in the connection strength and transmission efficiency at pre-existing synapses [19, 20]. It serves as the foundation for memory and cognitive processes and can be facilitated by synaptic functional modification, such as, changes in synaptic strength or structure [21, 22]. The central nervous system’s (CNS) ability to absorb and retain information is directly hampered by the decline in synaptic connection strength and synaptic transmission efficiency, which impairs cognitive function [23, 24]. A wealth of evidence has unequivocally demonstrated the indispensability of brain-derived neurotrophic factor (BDNF) in the modulation of synaptic plasticity in neurons, which enhances synaptic transmission, transmitter release and new synaptic sprouting [25–28]. Its expression is regulated by the signal of cyclic adenosine monophosphate (cAMP) response element binding protein (CREB), a pivotal transcription factor implicated in learning and memory [29, 30].

Despite some studies suggesting a potential link between neuroinflammation and neuropsychiatric disorders following CHF [31, 32], the precise role of synapses in cognitive impairment after CHF remains elusive in the context of the neuroinflammatory microenvironment. Therefore, we aimed to probe the impact of CHF on cognitive function from a more microscopic perspective and hypothesized that CHF might disrupt synaptic plasticity regulation via neuroinflammation, ultimately resulting in cognitive impairment.

## MATERIALS AND METHODS

### Animals and surgeries

The experimental processes followed the National Institutes of Health (NIH Publication No. 85-23, revised 1996) and were sanctioned by the Committee of Experimental Animal Administration of Dongzhimen Hospital of Beijing University of Chinese Medicine (approval No. 22-12). Male Sprague–Dawley rats (220 ± 10 g, SPF) were procured from Vital River Laboratory Animal Technology Co., Ltd., Beijing, China. All animals were habituated in a climate-controlled room with consistent temperature and humidity, as well as a 12-hour light/dark cycle, and they had unlimited access to water and standard laboratory food. Rats were anesthetized intraperitoneally with 1% pentobarbital (50 mg/kg body weight) and connected to ventilators (Alcott, ALC-V8) under isoflurane maintenance. CHF models were induced through coronary artery occlusions of the left anterior descending branch, as previously described [33]. On the rib cage of the rat, a 1.5 cm incision was made on the parasternal bone, specifically between the 3^rd^ and 4^th^ ribs. The left coronary artery was ligated at the 3^rd^ intercostal incision in the left anterior thoracic region of the rat, approximately 1 to 2 mm below the junction of the pulmonary cone and the left auricle. The thoracic cavity was subsequently closed using layer-by-layer sutures after rapid hemostasis. The identical surgery was performed on those in the Sham group without ligation. CHF models were confirmed by a surface electrocardiogram, which showed an arch-dorsal elevation of the ST segment in more than 2 limb leads or thoracic leads. The rats that survived 24 hours after the surgery, displaying pathological Q-waves ≥ 6 on the electrocardiogram, were included as CHF models [34].

### Echocardiography and hemodynamic evaluations

Cardiac function was assessed through the utilization of echocardiography and hemodynamics, as previously documented [35, 36]. Following the administration of anesthesia to the rats, the 21-MHz phased-array probe (Vevo 2100, VisualSonics Inc, Canada) was used to measure the left ventricular ejection fraction (LVEF) and fractional shortening (LVFS) values. Three to five cardiac cycles were recorded during each measurement, and the average values were obtained through three repetitions.

A hemodynamic evaluation was carried out by bluntly separating the anterior cervical muscle group with a 4 cm midline neck incision to expose the right common carotid artery. A pressure transducer was then inserted into the artery and advanced into the heart to measure the left ventricular systolic pressure (LVSP), left ventricular end-diastolic pressure (LVEDP), the maximal upstroke velocity of left ventricle (+dp/dt_max_), and the maximal descent velocity of left ventricle (−dp/dt_max_), which were recorded by a bio-function experiment system (Biopac, USA).

### Behavior tests

We evaluated spatial learning and memory function using the Morris water maze (MWM) platform (XR-XM101, Shanghai, China) in accordance with a previous study [37]. The test consists of an acquisition trial and a probe trial. During the acquisition trial, rats were randomly placed in the water from one of the three quadrants, excluding the third quadrant where the platform was located. The experiment was terminated upon the rats successfully locating the platform. In the event that the rats were unable to locate the platform within a 60-second timeframe, they were guided to it and allowed to remain on it for a duration of 10 seconds. Over a period of five consecutive days, the duration of time that rats spent in searching for the platform was documented. The platform was removed after the acquisition experiment, and the probe trial was then carried out. Except for the quadrant where the platform was initially positioned, rats could enter the pool from any of the remaining three quadrants. The number of times that rats crossed the platform area within a 60-second period was recorded.

We used the SMART V3.0 software (Rayward, Shenzhen, China) to perform the open field test (OFT) and evaluate the locomotor activity and exploratory behavior, following the methodology previously described [32]. Prior to the commencement of the experiment, the operator engaged in a daily 10-minute session of rat stroking for a duration of 5 days, with the intention of acquainting the rats with the operator’s scent. During the experiment, the rats were allowed to acclimate to the experimental room for one hour to reduce the impact of extraneous environmental factors. Subsequently, a solitary rat was situated within a clean, open box with a black interior measuring 100 cm × 100 cm × 40 cm, under subdued lighting conditions. The trajectory of the rat was recorded to analyze the distance travelled, the total resting time, the number of times it entered the central area, and the time it stayed in the central area within 5 minutes. Each rat was measured only once, and the open box was thoroughly cleaned after the measurement before the next observation.

### Nissl’s staining

Continuous coronal brain sections of 5 μm thickness were prepared after dehydration, clearing, and paraffin embedding. The 5 μm brain slices were immersed in Nissl’s staining solution (Toluidine Blue) (Servicebio, Wuhan, China) for 15 minutes. The sections were then rinsed with a phosphate buffer, dehydrated in ethanol, and cleared in xylene. Images were acquired by 3Dhistech Pannoramic Scan (3DHISTECH, Hungary). ImageJ software was used to analyze Nissl bodies in the CA1, CA3, and DG regions located in the hippocampus in a blinded manner.

### Transmission electron microscopy (TEM)

The hippocampal tissues were sectioned into strips measuring no more than 1 mm × 1 mm × 1 mm and expeditiously immersed in a 2.5% glutaraldehyde solution for a duration of 24 hours. Following a rinse in phosphate buffer and fixation in 1% osmium tetroxide, a sequence of dehydration stages using varying concentrations of acetone was executed. Subsequently, the tissue was implanted in epoxy resin, and 80 nm sections were produced and stained with uranyl acetate and lead citrate. Ultimately, each sample was cut into at least three blocks of ultrathin slices and analyzed using a transmission electron microscope (Hitachi, Japan).

### Immunofluorescence staining

Immunostaining was conducted to label reactive microglia and astrocytes using Iba1 and GFAP markers, respectively. Brain tissue samples were embedded in paraffin, and then continuous coronal sections of 5 μm thickness were prepared. Following PBS washing, antigen repair was performed for 20 minutes, and endogenous peroxidase activity was reduced by immersing the sections in 3% hydrogen peroxide for 20 minutes. After being blocked, the sections were incubated with diluted polyclonal rabbit anti-Iba1 (1:200, Wako, Japan) and anti-GFAP (1:300, Proteintech, China) antibodies. An Alexa Fluor 488-conjugated goat anti-rabbit IgG (Proteintech, China) was added as a secondary antibody and incubated for an hour at 37°C to stimulate immunoreactivity. DAPI (Boster Bio, USA) was used to counterstain the nuclei. A fluorescence microscope (×200 magnification) (Leica, Germany) was used to capture the images.

### Golgi staining and analysis

#### Golgi staining

The brains were expeditiously extracted without perfusion and subsequently immersed in a 4% PFA solution for 48 hours. The Golgi Stain Kit (Servicebio, Wuhan, China) was employed for the staining of brain tissues. The staining process was conducted in adherence to the material safety guidelines and the manufacturer’s manual. A set of 200-μm-thick hippocampal sections was sliced, and panoramic images of the brain tissue were captured using the 3Dhistech Pannoramic Scan (3DHISTECH, Hungary). Three neuronal cells with a well-defined structure and clear branches in the hippocampal region were selected for observation in each slice.

#### Sholl analysis

The Sholl analysis plugin in ImageJ software was used for the analysis of dendritic branch length. The number of crossings between the dendrites and the concentric circles was counted using the cell body of the neuron as the center of the circle and concentric circles with a separation of 10 μm. The overall branching and length of the dendrites were determined by counting the junctions. In addition, the density of the dendritic spines was examined. We obtained densities for each cell in segments that were at least 30 μm long. The classification of dendritic spines includes four distinct types: mushroom, stubby, long, and filopodial-like. Based on their shapes, mushroom and stubby dendritic spines are considered mature, whereas the remaining types are considered immature. The density of mature and immature spines was determined as the number of spines per 10 μm of dendritic segment. Since the spines that are hidden by other dendrites were not corrected, the actual density may be underestimated.

### Cell culture and treatment

BV2 microglial cells and HT22 mouse hippocampal cells (sourced from the National Biomedical Experimental Cell Resource Bank in Beijing, China) were cultured in high-glucose Dulbecco’s modified Eagle’s medium (DMEM) (Solarbio, Beijing, China) with 10% fetal bovine serum (Gibco, New York, USA) under conditions of 37°C and 5% CO_2_. Lipopolysaccharide (LPS) (Solarbio, Beijing, China) was dissolved in dimethyl sulfoxide to prepare a 1 mg/mL stock solution, which was then diluted in DMEM to the desired concentrations. BV2 cells were exposed to 1 μg/mL LPS for 24 hours in subsequent experiments.

### The cell-cell interaction models

We used a conditioned medium to establish a co-culture system using BV2 and HT22 cells to investigate neuroinflammation-mediated neuronal damage.

### Preparation of BV2-conditioned medium

Cell culture supernatants were obtained from BV2 cells that had reached 90% confluence and centrifuged for 5 minutes at 1200 rpm in a 15 mL tube. The clear supernatant was used for subsequent experiments or assays immediately or stored at −80°C. We used BV2 cell conditioned medium (C-CM) without LPS as a control. LPS-stimulated (1 μg/mL) BV2 cell conditioned medium, 24 hours post-LPS stimulation was used as model conditioned medium (L-CM).

### HT22 was incubated with BV2 cell-conditioned medium

HT22 cells were planted at a density of 3 × 10^5^ cells per well in 6-well culture plates or at a density of 8 × 10^3^ cells per well in 96-well culture plates, respectively. After adherence, the conditioned medium of each group was added and incubated for 24 hours. The HT22 cells were examined by phase-contrast microscopy (Olympus, Japan) to determine their morphological changes.

### Detection of nitric oxide (NO)

BV2 microglial cells were seeded in 96-well plates at a density of 1.0 × 10^4^ cells/well and subjected to treatment with 1 μg/mL LPS for 24 hours. We collected the culture supernatant and determined the concentration of NO using an NO assay kit according to the manufacturer’s instructions (Beyotime, Shanghai, China). With a microplate reader (Rayto, Shenzhen, China), the absorbance was measured at 540 nm.

### Cell viability assay of HT22

The present study employed the Cell Counting Kit-8 assay to assess the impact of microglial activation on HT22 cell viability. Specifically, BV2 cells were subjected to LPS stimulation (1 μg/mL) for 24 hours, after which co-culture was established for an additional 24 hours. Then, each well received 10 μL of the Cell Counting Kit-8 reagent (Aoqing, Beijing, China), which was then incubated for 3 hours at 37°C. A microplate reader (Rayto, Shenzhen, China) was then used to measure the absorbance at 450 nm.

### Enzyme-linked immunosorbent assay (ELISA)

Blood was acquired from abdominal veins and centrifuged at 3000 rpm, 4°C for 15 minutes to collect serum. Brain natriuretic peptide (BNP), N-terminal pro-B-type natriuretic peptide (NT-proBNP), angiotensin II (Ang II), cardiac troponin T (cTnT), soluble ST2 (sST2) levels in the serum were measured to assess the severity of CHF using commercially available ELISA kits (Dogesce, Beijing, China). We separated and obtained hippocampal tissue from each group to prepare tissue homogenate. The homogenate was centrifuged for 10 minutes at 4°C and 3500 rpm to separate the supernatant. IL-1β, TNF-α, interleukin-10 (IL-10), and BDNF in the hippocampus were detected using commercially available ELISA kits (Dogesce, Beijing, China). The culture medium for BV2 cells in each culture hole was collected, and the supernatant was taken after centrifugation at 3000 rpm, 4°C for 15 minutes. According to the manufacturer’s instructions, the levels of TNF-α and IL-1β in the supernatant were measured.

### Western blot analysis

Iba1 and GFAP expression levels were measured to detect reactive microglia and astrocytes. The levels of postsynaptic density protein-95 (PSD95), Synapsin I, BDNF, PDE4, CREB, and p-CREB were measured in hippocampal tissues or HT22 cells. Hippocampal tissues and HT22 cells were isolated and subjected to total protein extraction using RIPA lysis buffer (Beyotime, Shanghai, China). The BCA protein assay kit (Beyotime, Shanghai, China) was used to quantify protein. The samples were denatured with loading buffer (Lablead, Beijing, China), and then kept at −80°C until further use. The proteins were transferred onto nitrocellulose filter membranes (Pall Corporation, USA) after being separated on SDS-PAGE gels (30 μg/lane). A 5% skim milk solution containing 0.05% Tween 20 in TBST was used as a blocker for 2 hours, followed by incubation with antibodies against PSD95 (1:1000, CST, USA), Synapsin I (1: 1000, CST, USA), Iba1 (1:1000, Proteintech, China), GFAP (1:5000, Proteintech, China), BDNF (1:1000, Abways, China), PDE4 (1:1000, Immunoway, China), CREB (1:1000, Proteintech, China), p-CREB (1:1000, CST, USA), and β-actin (1:5000, Proteintech, China) in TBST overnight at 4°C with gentle shaking. After rinsing, horseradish peroxidase-conjugated secondary antibodies (1:8000, Proteintech, China) were added and incubated for 2 hours. We visualized the immunoblotting results using an enhanced chemiluminescence detection system (Lablead, Beijing, China) and measured optical density by using ImageJ.

### 16S rRNA sequencing

The fecal samples were collected and transferred to Eppendorf tubes. The frozen samples were subjected to processing by Shanghai Majorbio Bio-Pharm Technology Co., Ltd., (Shanghai, China). The DNA extraction and purification process involved the utilization of the cetyltrimethylammonium bromide/sodium dodecyl sulfate method and 1% agarose gels. Through amplification of the targeted sequencing regions and PCR utilizing TransGen AP221-02 (TransStart FastPfu DNA Polymerase), specific primers including barcodes were synthesized. PCR products from the same sample were combined and analyzed via 2% agarose gel electrophoresis, with the AxyPrepDNA Gel Recovery Kit (AXYGEN, USA) being employed to recover PCR products. PCR products were detected and quantified using the QuantiFluor^™^-ST Blue Fluorescence Quantification System (Promega, USA), with preliminary quantification results from electrophoresis being taken into consideration. A paired-end sequence was performed on an Illumina MiSeq platform with purified amplicons pooled in equimolar amounts.

### Bioinformatic analysis for intestinal flora

Data analysis was performed using the Majorbio I-Sanger Cloud Platform. The intestinal flora community changes among the three groups were examined through the evaluation of OTUs, community structure, α-diversity, and β-diversity. The Shannon, Simpson, and Heip indices were employed as α-diversity indices. The β-diversity was analyzed through simple distances obtained by principal coordinates analysis (PCoA). Based on linear discriminant analysis effect size (LEfSe), microbiota alterations were evaluated between two groups. Furthermore, the relationship between microbiota and cytokines was illustrated through Spearman analysis.

### Statistical analysis

With the help of the GraphPad Prism version 8.0 software (GraphPad Software, La Jolla, CA, USA), statistical analyses and visualization were conducted using the student’s two-tailed *t*-test for two groups and one-way analysis of variance (ANOVA) followed by Dunnett’s test for three groups. The quantitative data were presented as Mean ± SEM. The statistical significance criterion was set at *P* < 0.05 for analyzing all the results among the groups.

### Availability of data and materials

The datasets analyzed during the current study are available from the corresponding author on reasonable request.

## RESULTS

### Determination of CHF induced by surgical myocardial infarction

**Figure 1:**
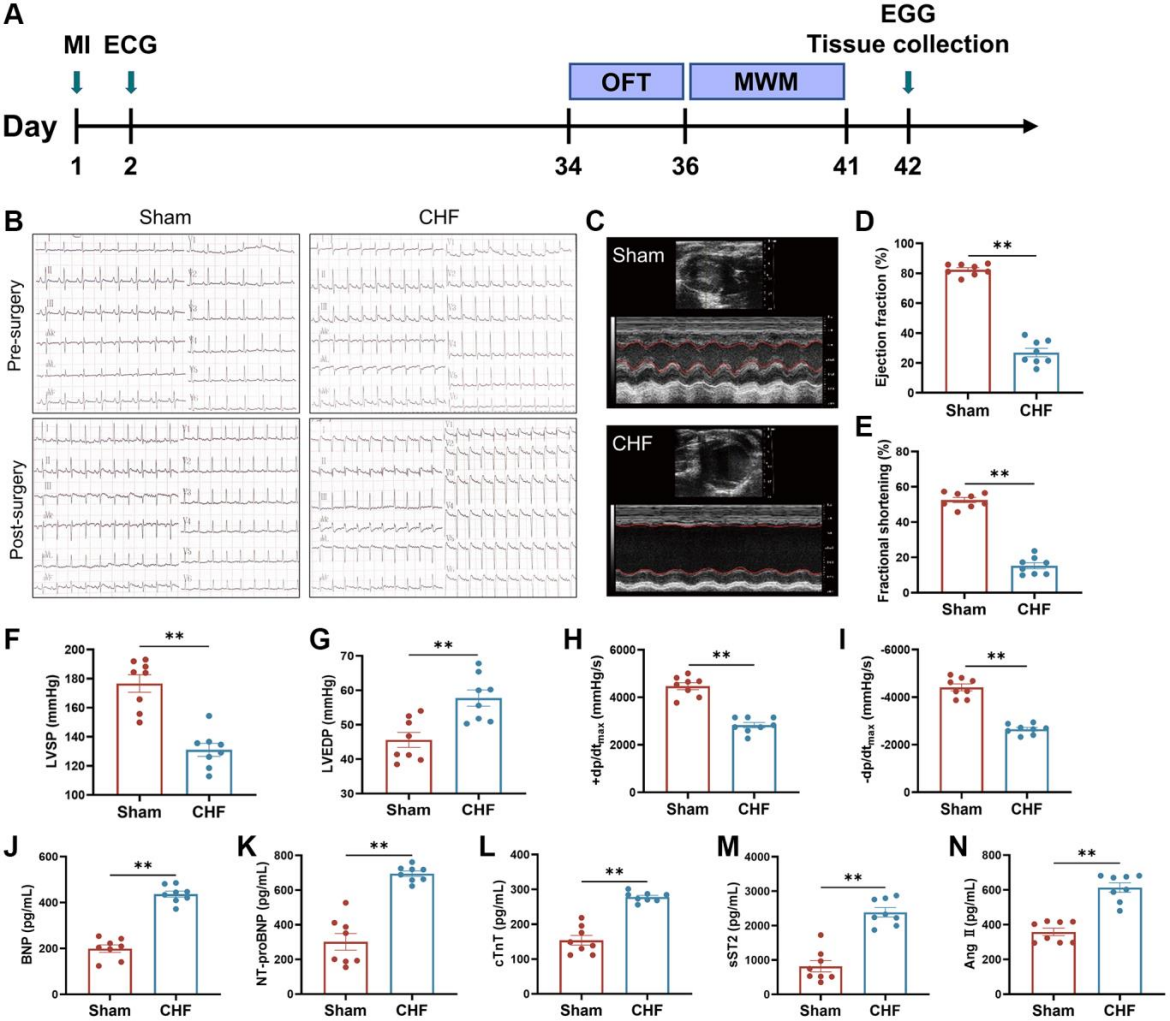
**The cardiac function and hemal physiological and biological indices in CHF rats.** (**A**) Animal experiment timeline. (**B**) Representative electrocardiograms at 24 hours before and after surgery. (**C**–**E**) Representative M mode echocardiograms of Sham and CHF rats (**C**) and quantifications of left ventricular ejection fraction (LVEF) (**D**), left ventricular fraction shortening (LVFS) (**E**). (**F**–**I**) Quantifications of cardiac hemodynamic parameters, including left ventricular systolic pressure (LVSP) (**F**), left ventricular end-diastolic pressure (LVEDP) (**G**), +dp/dtmax (**H**), −dp/dtmax (**I**). (**J**–**N**) The BNP (**J**), NT-proBNP (**K**), cTnT (**L**), sST2 (**M**) and Ang II (**N**) levels in serum were measured by ELISA. Data are presented as the Mean ± SEM; ^*^P < 0.05, and ^**^P < 0.01 vs. Sham; *n* = 8.

Figure 1A presents the timeline of the experiment. The results of electrocardiography, displaying pathological seven Q-waves, confirmed the successful construction of the MI models (Figure 1B). In order to examine the cardiac function of rats with CHF six weeks after MI, echocardiography was utilized to measure LVEF and LVFS to assess myocardial contractility. The results showed that the CHF group exhibited a significant decrease in LVEF and LVFS compared to the Sham group, suggesting that MI had a detrimental effect on cardiac function (Figure 1C–1E). These findings were further supported by changes in hemodynamic parameters, as evidenced by a significant decrease in LVSP, +dp/dt_max_ and −dp/dt_max_, respectively, in CHF rats, and a significant increase in LVEDP (Figure 1F–1I).

Cardiac biomarkers, namely BNP, NT-proBNP, and cTnT, are frequently employed as independent predictors for the diagnosis of CHF, with their levels being elevated in patients with the condition [38]. The severity of CHF was evaluated by detecting the levels of BNP, NT-proBNP, and cTnT in serum using ELISA kits. The CHF group exhibited a significant increase in the levels of BNP, NT-proBNP, and cTnT when compared to the Sham group (Figure 1J–1L). sST2, a sensitive indicator of myocardial fibrosis and ventricular remodeling, is produced by cardiomyocytes in response to adverse stimuli [39]. Ang II is a hormone that promotes vasoconstriction and increases cardiac hypertrophy [40]. CHF triggered an increase in sST2 and Ang II levels (Figure 1M, 1N). These results demonstrated that our surgical procedure effectively and sufficiently caused cardiac dysfunction and CHF in rats.

### CHF induces hippocampus-dependent learning and memory dysfunction

**Figure 2:**
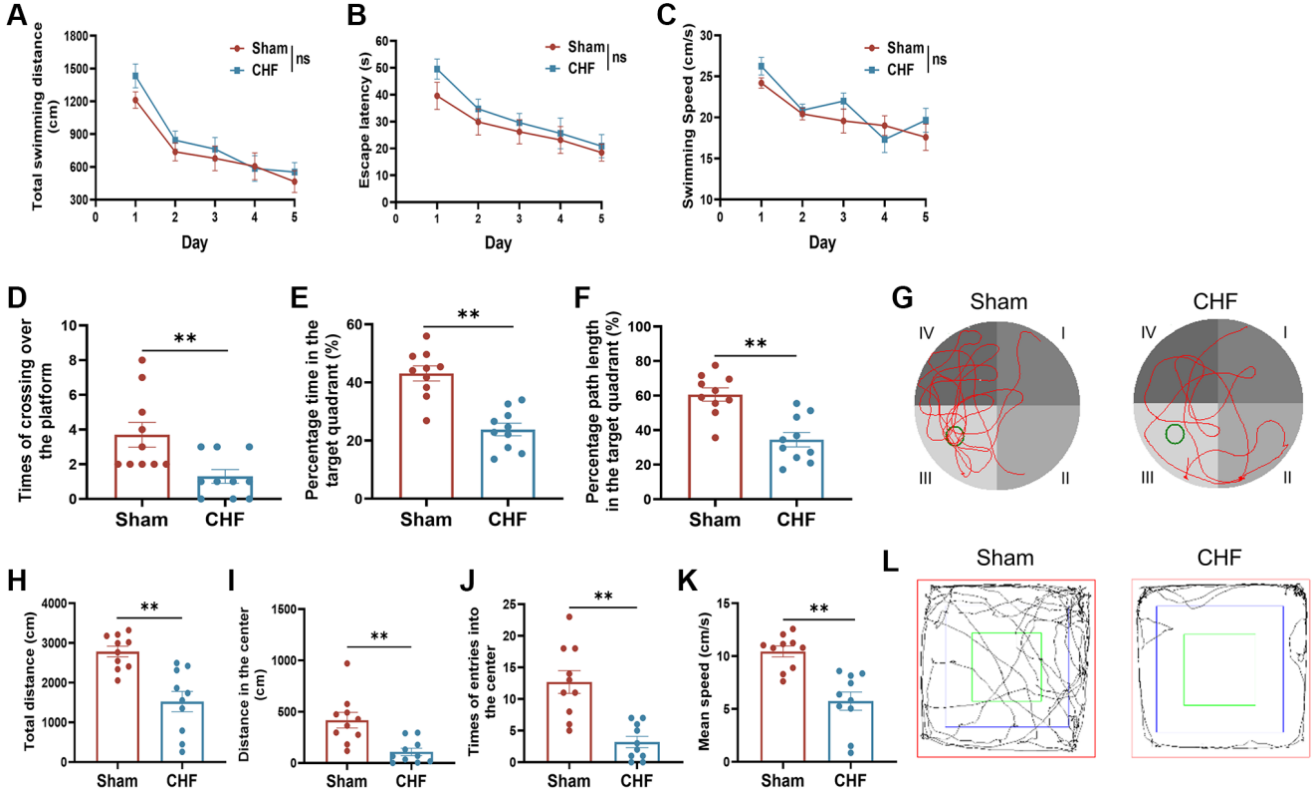
**CHF induces poor performance in cognitive behaviors in CHF rats.** (**A**–**C**) MWM acquisition trial, including total average swimming distance (**A**), escape latency period for rats to find the underwater platforms (**B**), and average swimming speed (**C**). (**D**–**F**) MWM probe trial, including times of crossing over the platform (**D**), percentage of the time (**E**), and distance (**F**) spent in the target quadrant. (**G**) Representative paths of Sham and CHF rats in the spatial probe test. (**H**–**K**) The parameters of the open field test, including total distance (**H**), distance in the center (**I**), times of entries into the center (**J**), and mean speed (**K**). (**L**) Representative paths of Sham and CHF rats in the open field test. Data are presented as the Mean ± SEM; ^*^P < 0.05, and ^**^P < 0.01 vs. Sham; *n* = 10.

The MWM test was carried out to assess the ability of learning and memory in CHF rats. In an acquisition trial, a decline in escape latency time and total swimming distance was observed from the 1^st^ day to 5^th^ day, but there was no significant difference between the Sham and CHF groups (Figure 2A, 2B). There were no significant differences in swimming speed between the two groups (Figure 2C). However, during the probe trial, the CHF group displayed a decline in the number of times crossing the platform, the percentage of time spent in the target quadrant, and the percentage of path length in the target quadrant compared to the Sham group (Figure 2D–2G). These results manifested that spatial memory was significantly impaired in CHF rats.

The OFT results demonstrated significant reductions in total distances, distances in the center, number of entries into the center, and average speed of CHF rats when compared to the Sham group (Figure 2H–2L). This suggests that CHF might reduce the locomotor activity of the rats to a certain extent. The results of the behavioral tests above revealed that CHF may be a distraction, which impairs learning and memory function.

### CHF activates neuroinflammatory response via microglia and astrocytes in the hippocampus

**Figure 3:**
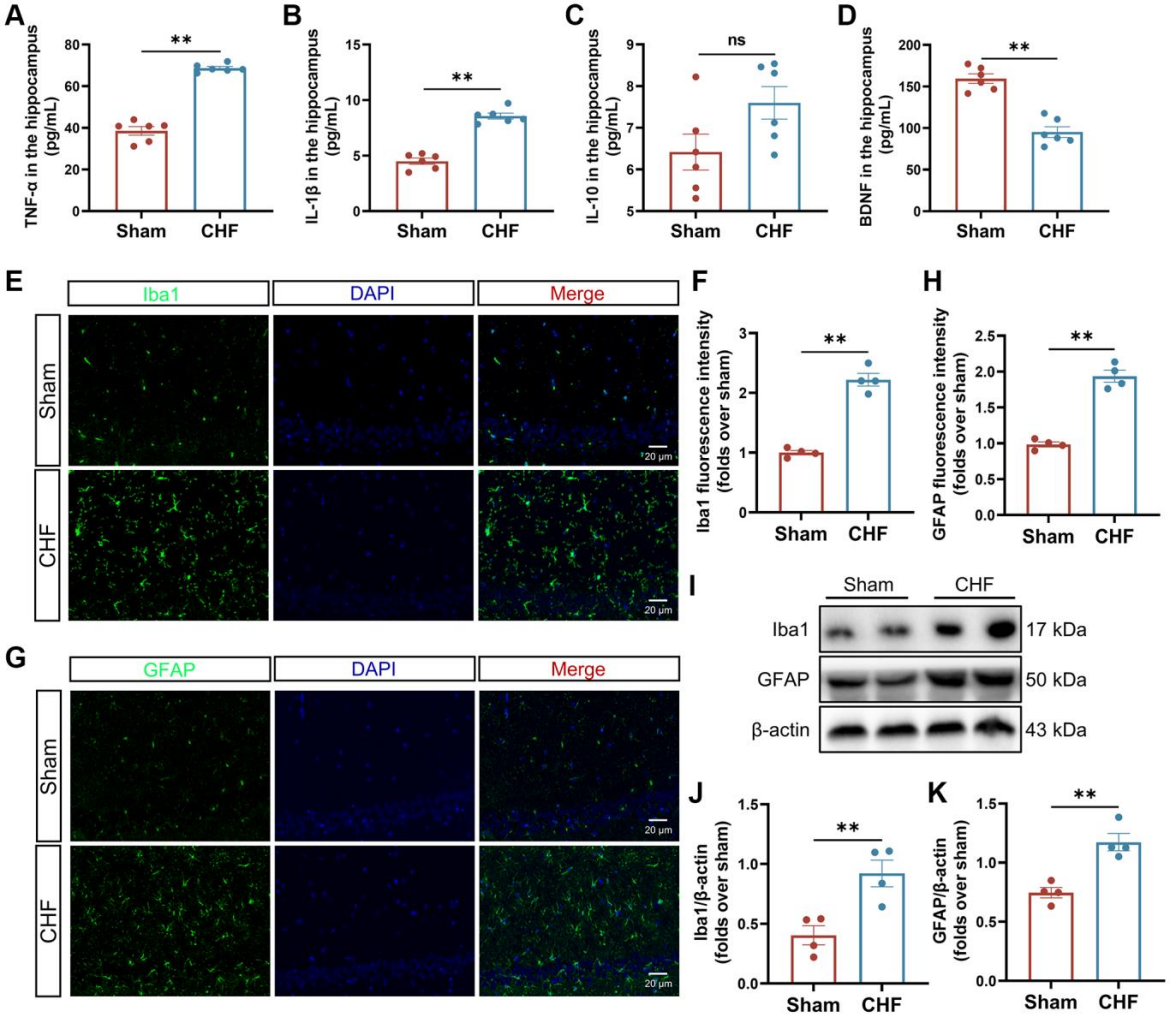
**CHF triggers the inflammatory responses via the activation of microglia and astrocytes.** (**A**–**D**) The content of TNF-α (**A**), IL-1β (**B**), IL-10 (**C**), and BDNF (**D**) in the hippocampus was detected by ELISA *n* = 6. (**E**, **F**) Iba1 immunofluorescence staining in the hippocampus and their quantitative analysis. Green: Iba1; Blue: DAPI. Scale bars: 20 μm *n* = 4. (**G**, **H**) GFAP immunofluorescence staining in the hippocampus and their quantitative analysis. Green: GFAP; Blue: DAPI. Scale bars: 20 μm *n* = 4. (**I**–**K**) Representative Western blots (**I**) and quantification data of Iba1 (**J**) and GFAP (**K**). β-actin was used as a loading control. Data are presented as the Mean ± SEM; ^*^P < 0.05, and ^**^P < 0.01 vs. Sham; *n* = 3.

Elevated levels of proinflammatory cytokines circulate in CHF patients with reduced ejection fraction. We further measured the content of inflammatory cytokines in the hippocampal region. Our results indicated that CHF significantly increased the secretion of IL-1β and TNF-α in the hippocampus when compared to the Sham group (Figure 3A, 3B), with a slight elevation in IL-10 (Figure 3C), suggesting that CHF may stimulate inflammatory responses in the hippocampus. BDNF is critical for mediating long-term potentiation and depression, promoting neuronal synaptogenesis, and enhancing hippocampal synaptic plasticity [41]. The level of BDNF in the CHF group decreased significantly compared to the Sham group (Figure 3D).

Microglia and astrocytes are the main sources of inflammatory response in the CNS. The assessment of microglia and astrocytes was conducted using both immunofluorescence and Western blot analysis. The Sham group exhibited a low intensity of fluorescence signal in Iba1-positive microglia and GFAP-positive astrocytes, whereas the CHF group displayed an increase in the Iba1 and GFAP signals (Figure 3E–3H). These findings were further supported by Western blot analysis (Figure 3I–3K), which revealed a significant upregulation of Iba1 and GFAP proteins in the CHF group, implying that microglia and astrocytes might be activated by CHF.

### CHF induces the loss and damage of neurons in the hippocampus

**Figure 4:**
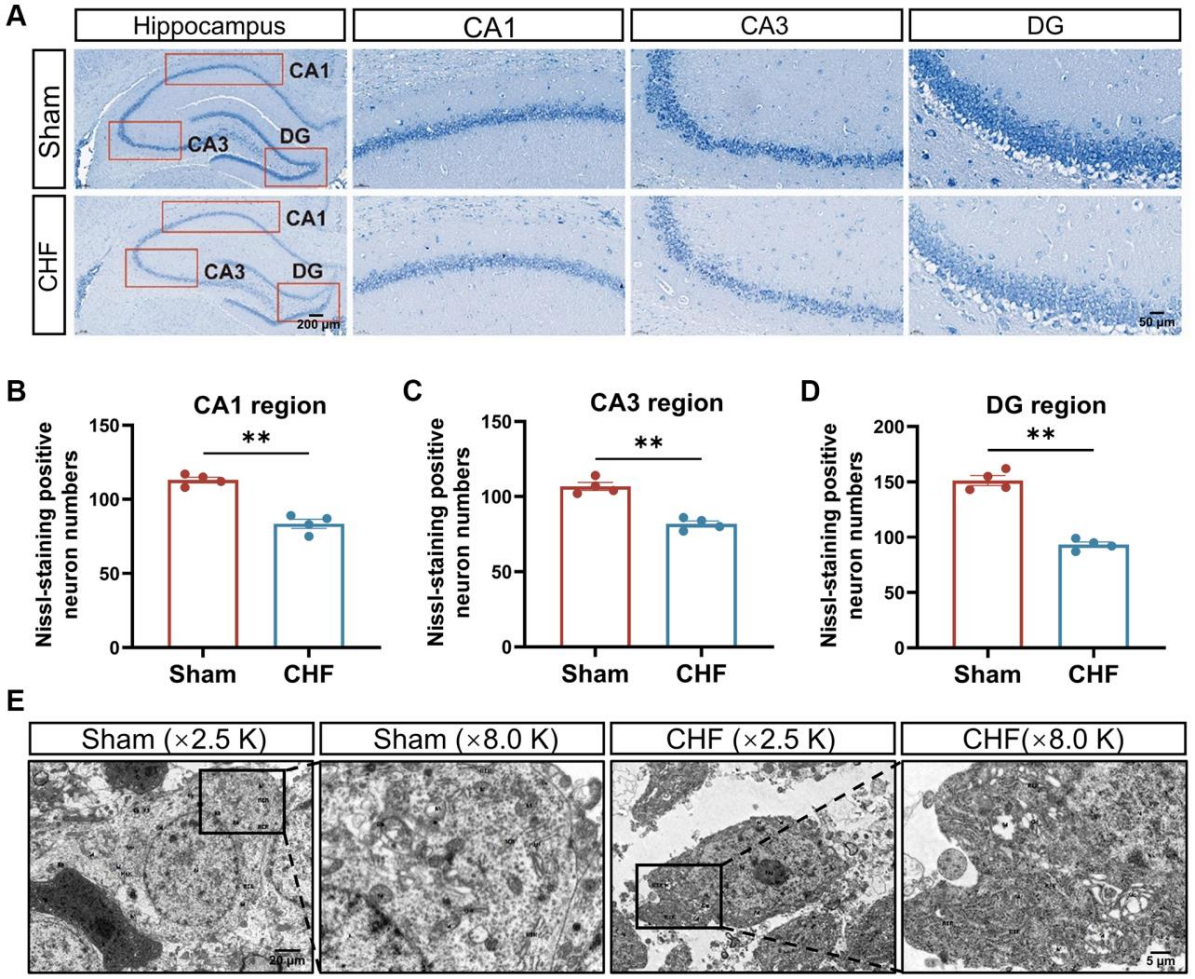
**CHF causes the loss of Nissl bodies and ultrastructural damage of neurons in the hippocampus.** (**A**) Representative images of Nissl’s staining. (**B**–**D**) Quantifications of Nissl bodies in CA1, CA3, DG regions in the hippocampus, respectively *n* = 4. (**E**) Representative ultrastructural images of neurons shown at magnifications of ×2500, scale bars: 20 μm; ×8000, scale bars: 5 μm. Data are presented as the Mean ± SEM; ^*^P < 0.05, and ^**^P < 0.01 vs. Sham; *n* = 3.

Nissl bodies synthesize structural proteins, neurotransmitter-synthesizing enzymes, and neuromodulatory peptides needed for organelle renewal [42]. Neuronal damage can lead to a decrease, disintegration, or complete loss of Nissl bodies. The Sham group exhibited densely arranged nerve cells with a copious amount of Nissl bodies in the cytoplasm. Conversely, the CHF group displayed a lighter cytoplasm and a slight reduction in the number of Nissl bodies in the CA1, CA3, and DG regions (Figure 4A–4D).

Transmission electron microscopy revealed severe edema of neurons in the CHF group compared to the Sham group, with localized cell membrane breakage, free organelles, large areas of low electron density edema, matrix lysis, and marked swelling of organelles. The mitochondria dissolved into vacuoles, and the matrix within the mitochondrial membrane dissolved, while cristae disappeared in the CHF group (Figure 4E).

### CHF alters synaptic ultrastructure, impairs synaptic plasticity, and disrupts the cAMP signaling pathway

**Figure 5:**
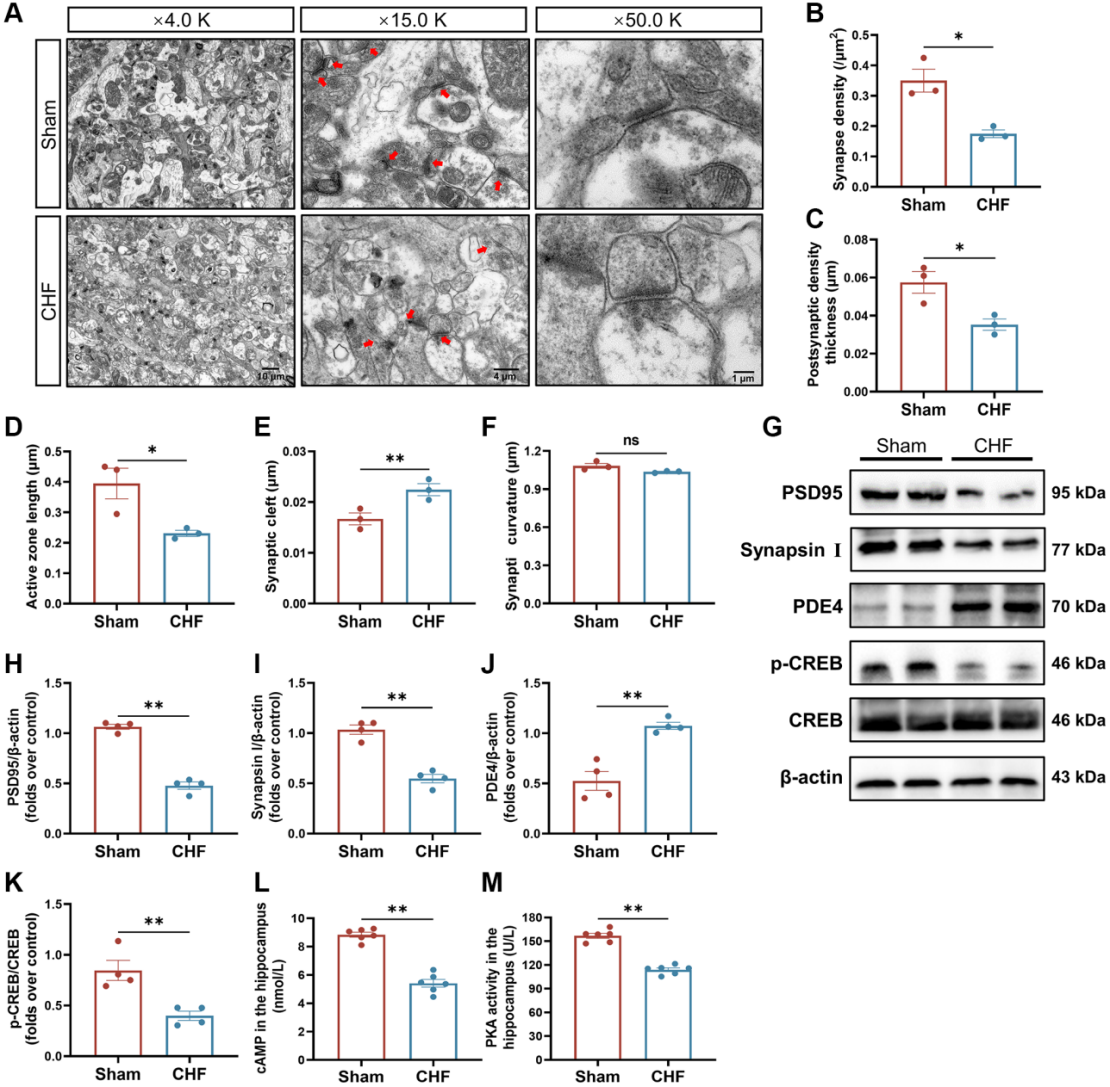
**CHF impairs synaptic ultrastructure in the hippocampus.** (**A**) Representative ultrastructural images of synapses shown at magnifications of × 4000, scale bars: 10 μm; × 15000, scale bars: 4 μm; × 50000, scale bars: 1 μm, respectively. (**B**–**F**) Quantifications of the synaptic interface, including synapse density (**B**), PSD thickness (**C**), active zone length (**D**), synaptic cleft (**E**), and synaptic curvature (**F**) *n* = 3. (**G**–**K**) Representative Western blots (**G**) and quantitative data of PSD95 (**H**), Synapsin I (**I**), PDE4 (**J**), p-CREB and CREB (**K**). β-actin was used as a loading control *n* = 3. (**L**) The content of cAMP in the hippocampus detected by ELISA. (**M**) The activity of PKA in the hippocampus detected by ELISA *n* = 6. Data are presented as the Mean ± SEM; ^*^P < 0.05, and ^**^P < 0.01 vs. Sham.

To definitively investigate the impact of CHF on synaptic plasticity, we examined the synaptic ultrastructural changes and the levels of crucial synaptic proteins (PSD95 and Synapsin I) in the hippocampus. There are distinct differentials in the anterior and posterior membranes of the synapse, called the presynaptic active region and postsynaptic density (PSD), respectively. The length of the presynaptic active region reflects the effective region of neurotransmitter release. The primary constituents of PSD consist of tubulin and actin, and its thickness is intricately linked to synaptic function, serving as a crucial morphological indicator of synaptic plasticity. Additionally, synaptic curvature plays a significant role in synaptic function. The synaptic cleft harbors numerous enzymes that degrade neurotransmitters, and its width is inversely correlated with the efficacy of neurotransmitter transmission. Synaptic density (red arrow) and interface parameters differed noticeably across the two groups (Figure 5A). Quantitative analysis of the hippocampal synapses showed that CHF rats had lower synaptic density (Figure 5B), thinner postsynaptic density thickness (Figure 5C), shorter active zone length (Figure 5D), and wider synaptic clefts (Figure 5E) compared to the Sham group. However, no discernible variations in synaptic curvature were observed between the two groups (Figure 5F). PSD95, a crucial marker of the postsynaptic membrane, is one of the most abundant proteins in the postsynaptic dense material. Synapsin I acts as a regulator of synaptic vesicle trafficking, controlling neurotransmitter release at the presynaptic terminal. In comparison to the CHF group, a significant reduction in both PSD95 and Synapsin I levels was observed (Figure 5G–5I).

Given that synaptic plasticity can be regulated by cAMP signaling, the content of cAMP, the activity of PKA, and their associated signaling proteins were evaluated. As noticed, the content of cAMP and the activity of PKA were decreased in CHF rats (Figure 5L, 5M). Additionally, the protein level of PDE4 was elevated in CHF rats (Figure 5G, 5J). In parallel, CHF rats showed lower expression of p-CREB protein (Figure 5G, 5K). The above morphology and protein data suggest that CHF might result in synaptic loss and impaired synaptic ultrastructure through the PDE4/cAMP/PKA/CREB pathway in the hippocampal region.

### CHF impedes the growth of dendrites and dendritic spines in the hippocampus

**Figure 6:**
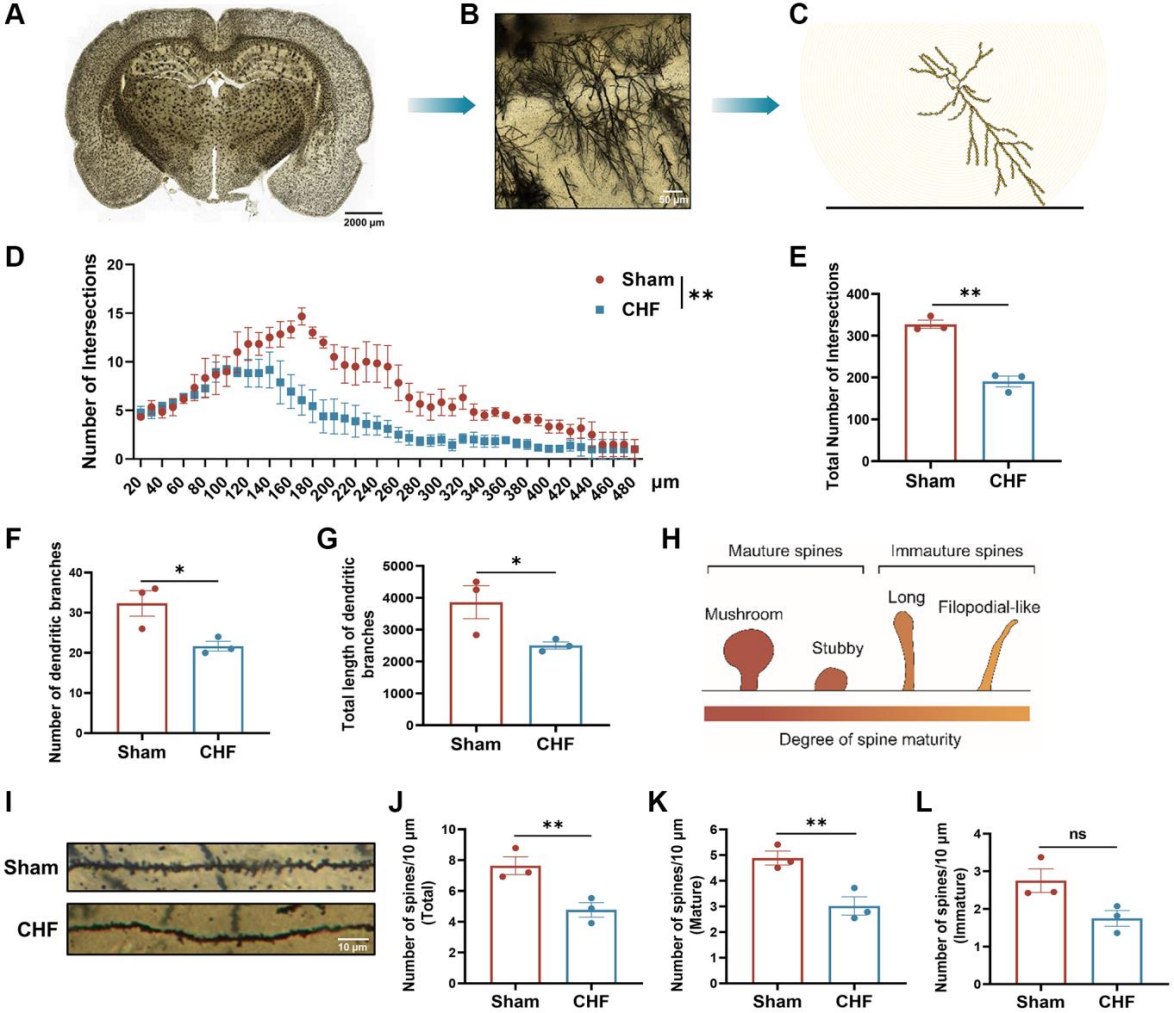
**CHF imperils the dendrites and dendritic spines in the hippocampus.** (**A**–**C**) Examples of reconstructed neurons showing dendritic complexity via Sholl analysis. Scale bars: 50 μm. (**D**) Dendritic intersection distribution at increasing distance from the cell body. (**E**–**G**) Total intersection points (**E**), dendritic branch numbers (**F**), and total dendritic branch lengths (**G**). (**H**) The classical classifications of spine morphology. (**I**) Representative examples of dendritic spines. Scale bar: 10 μm. (**J**–**L**) Density of dendritic spines, including the number of total spines/10 μm, the number of mature spines/10 μm, the number of immature spines/10 μm. Scale bars: 10 μm. Data are presented as the Mean ± SEM; ^*^P < 0.05, and ^**^P < 0.01 vs. Sham; *n* = 3.

The dynamic nature of dendritic spines is integral to structural synaptic plasticity, which is essential for cognition and memory [43]. The study analyzed various dendritic features, including the total number of intersections, the count of dendritic branches, and the total length of dendritic branches in the hippocampus so as to ascertain the impact of CHF on dendritic growth. Golgi staining was utilized to fill the dendritic shafts and spines of neurons (Figure 6A–6C). Sholl analysis indicated that CHF had a negative impact on the total number of intersections compared to the Sham group (Figure 6D, 6E). In relation to the total number of dendritic branches, CHF significantly decreased the number of dendritic branches when compared to the Sham group (Figure 6F). A noticeable decline in total dendritic length was also observed after CHF (Figure 6G).

Subtle changes in dendritic spine morphology and structure affect synaptic function, the connectivity patterns of neural circuits and cognitive behavior. We observed microstructural modifications in the morphology of dendritic spines in the hippocampus and employed the classical classification of spine morphology to determine the density of mature and immature spines (Figure 6H). Representative images of Golgi-stained dendritic spines from the hippocampal region are displayed (Figure 6I). Immature dendritic spines are believed to be linked to learning behaviors, while mature dendritic spines are associated with memory storage functions. The CHF group showed a statistically significant decline in total spine density compared to the Sham group (Figure 6J). Additionally, the density of mature spines was noticeably lower than that of the Sham group (Figure 6K). Also, the number of immature spines was marginally decreased by CHF (Figure 6L).

### Microglia of BV2 damage the synaptic plasticity of HT22 cells by regulating PDE4/cAMP/CREB/BDNF signals under LPS stimulation

**Figure 7:**
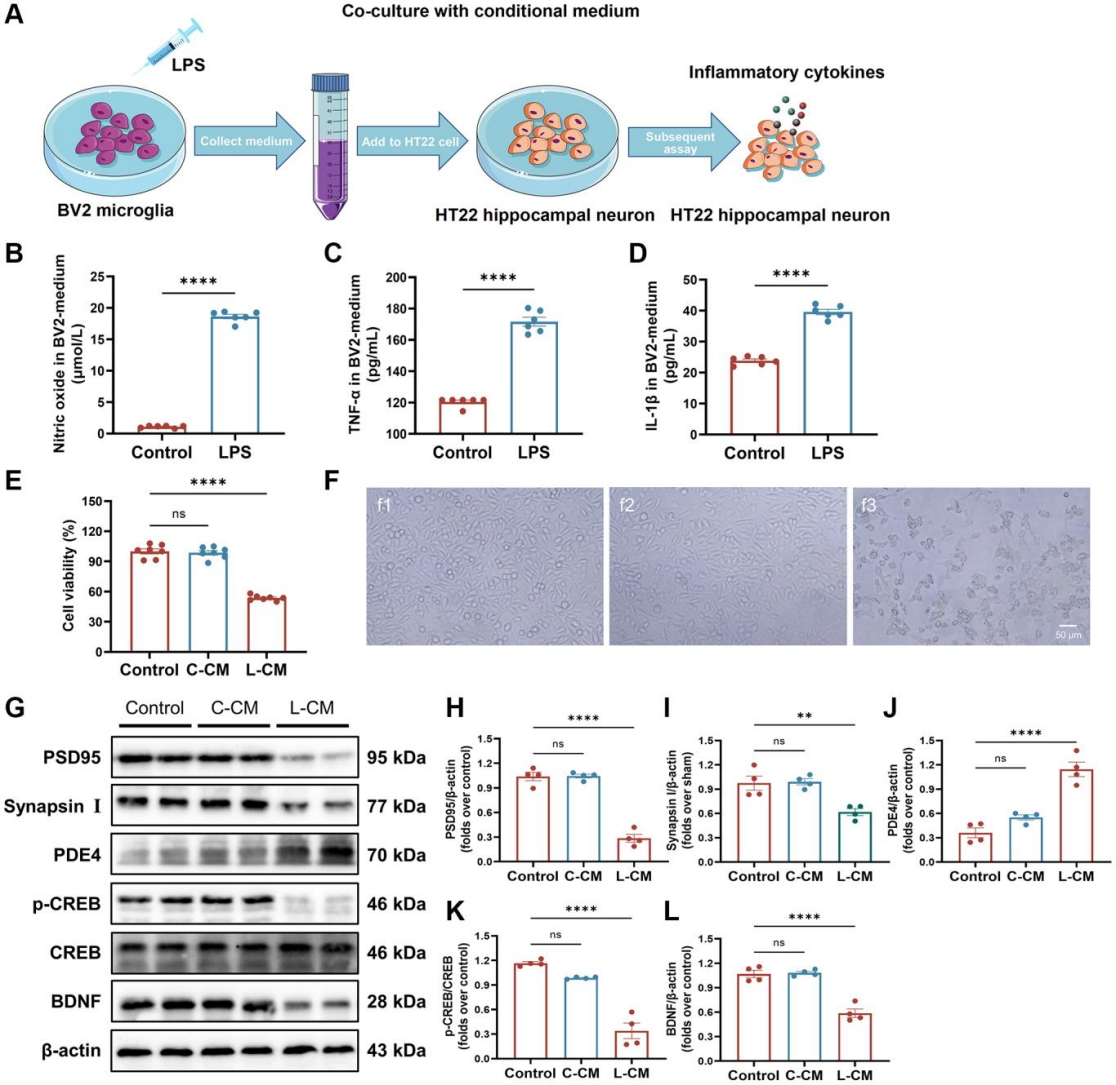
**BV2-induced inflammatory response damages neurons.** (**A**) Overview of a co-culture system of BV2 microglia and HT22 hippocampal neuronal cells. (**B**) The level of NO in BV2 cell-free supernatant measured with an NO kit. (**C**, **D**) The levels of TNF-α and IL-1β in BV2 cell-free supernatant detected with ELISA kits. (**E**) HT22 cell viability when cultured by BV2 cell-free supernatant was detected by Cell Counting Kit-8 kits. (**F**) Representative images of HT22 cells in each group were observed by a phase-contrast microscope. (**G**–**L**) Represent Western blots (**G**) and quantification data of PSD95 (**H**), Synapsin I (**I**), PDE4 (**J**), p-CREB and CREB (**K**), BDNF (**L**). β-actin was used as a loading control. Data are presented as the Mean ± SEM; ^*^P < 0.05, and ^**^P < 0.01 vs. Sham; *n* = 4.

To investigate the impact of neuroinflammation on neuronal growth and synaptic plasticity, a co-culture system consisting of BV2 microglia and HT22 hippocampal neurons was established to replicate the inflammatory microenvironment within the brain (Figure 7A). We precisely detected the levels of NO, TNF-α, and IL-1β in the culture medium of BV2 cells subsequent to LPS stimulation. As compared to the Sham group, the levels of NO, TNF-α, and IL-1β were found to be elevated (Figure 7B–7D). LPS-stimulated BV2 cells had an obvious adverse effect on the viability of HT22 cells (Figure 7E). The morphology of HT22 cells cultured in LPS-stimulated BV2 cell medium became out-of-shape, shrunken, fragmented, and even dead (Figure 7F). Furthermore, we determined the levels of Synapsin I, PSD95, and BDNF by Western blot. The treatment with LPS-stimulated BV2 medium led to decreased levels of Synapsin I, PSD95, and BDNF in HT22 cells (Figure 7G–7I, 7L). Furthermore, LPS-stimulated BV2 medium upregulated PDE4 and downregulated p-CREB, and there was no change in CREB protein (Figure 7G, 7J, 7K). Taken together, these results indicate that a neuroinflammatory microenvironment impairs neuronal growth and synapse proteins *in vitro* and is closely associated with the alteration of PDE4/cAMP/CREB/BDNF signaling.

### Intestinal flora dysbiosis induced by CHF is associated with cardiac function parameters, cytokines, and PDE4/cAMP/PKA/CREB signaling

**Figure 8:**
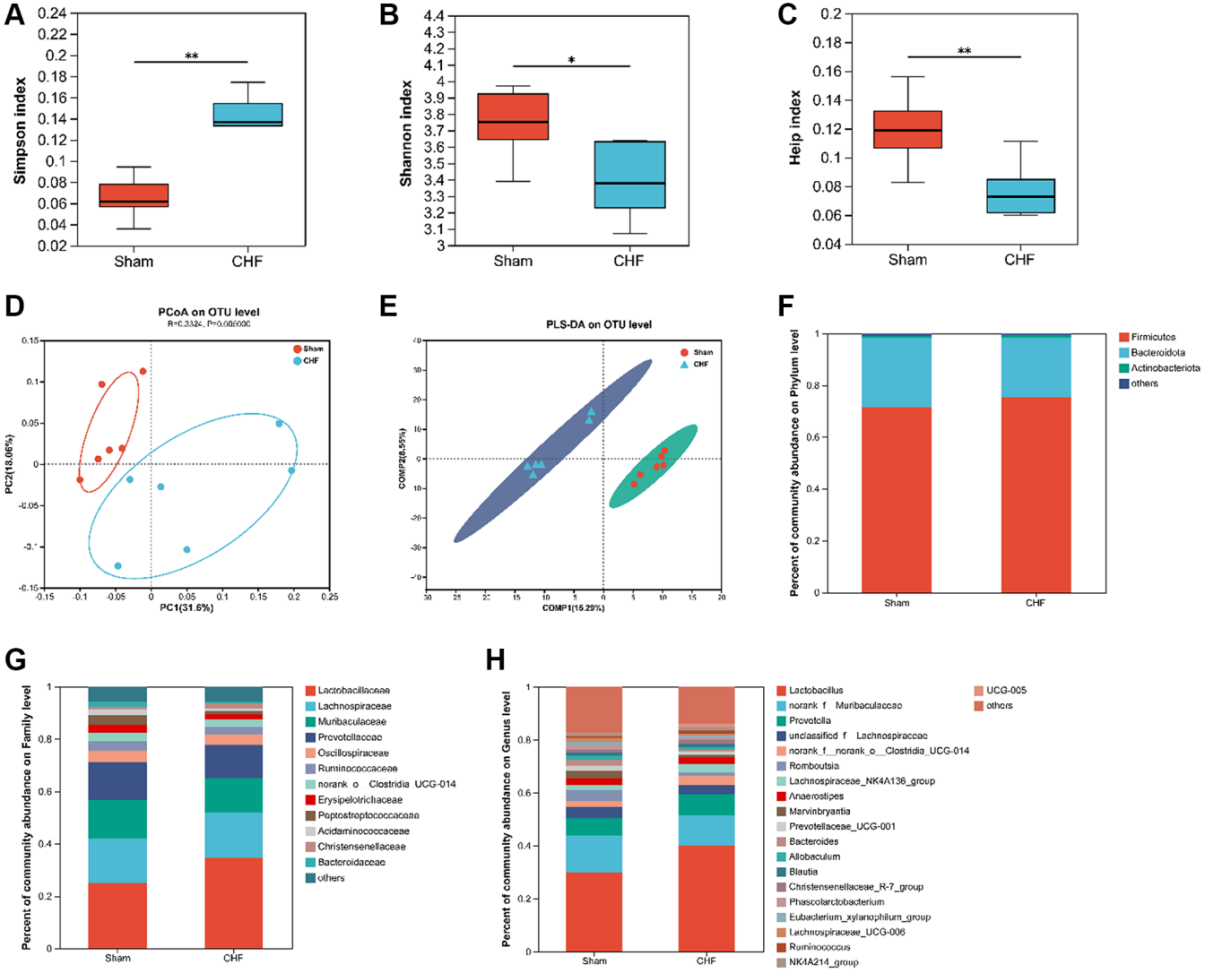
**The diversity and richness of intestinal flora in CHF rats.** (**A**–**C**) Decreased alpha diversity post-CHF based on Shannon (**A**), Simpson (**B**), and Heip (**C**) indices. (**D**) Beta diversity of intestinal flora differing between CHF and Sham groups. (**E**) PLS-DA score plots from 16S rRNA sequencing of fecal samples at OTU level. (**F**–**H**) Relative abundance of intestinal flora at the phylum level (**F**), family level (**G**), and genus level (**H**). Data are presented as the Mean ± SEM; ^*^P < 0.05, and ^**^P < 0.01 vs. Sham; *n* = 6.

Based on the limited evidence of organ crosstalk between gut microbiota and cardiocerebral disorders in CHF, we tried to detect modifications in gut microbiota to find clues to this crosstalk between heart and brain. 16S rRNA sequencing was used to analyze the intestinal flora of CHF rats. Alpha diversity reflects the diversity and richness of microbes. CHF decreased α-diversity based on Simpson, Shannon, and Heip indices (Figure 8A–8C), indicating that the gut microbiota diversity and richness of rats significantly declined after CHF. PCoA was used to visualize the divergence between the two groups in the community structure and diversity of intestinal flora to determine the potential effects of CHF. The majority of the Sham plots were grouped to the left, while the CHF plots were dispersed and not grouped in any particular location (Figure 8D). Clearly, CHF was grouped in a separate area from Sham (Figure 8E). Overall, the findings suggest a robust association between CHF and alterations in the composition of the intestinal flora. Specifically, at the phylum level, *Firmicutes* and *Bacteroidetes*, as well as *Actinobacteriota* were identified as the predominant phyla in the intestinal flora. There was no significant difference in the abundance of these three phyla between the Sham and CHF groups (Figure 8F). At the family level, the five primary families present in the intestinal flora were identified as *Lactobacillaceae*, *Lachnospiraceae*, *Muribaculaceae*, *Prevotellaceae*, and *Oscillospiraceae* (Figure 8G). The five major intestinal flora at the genus level were *Lactobacillus*, *norank_f_Muribaculaceae*, *Prevotella*, *unclassified_f_Lachnospiraceae*, and *norank_f_norank_o_Clostridia_UCG-014* (Figure 8H).

**Figure 9:**
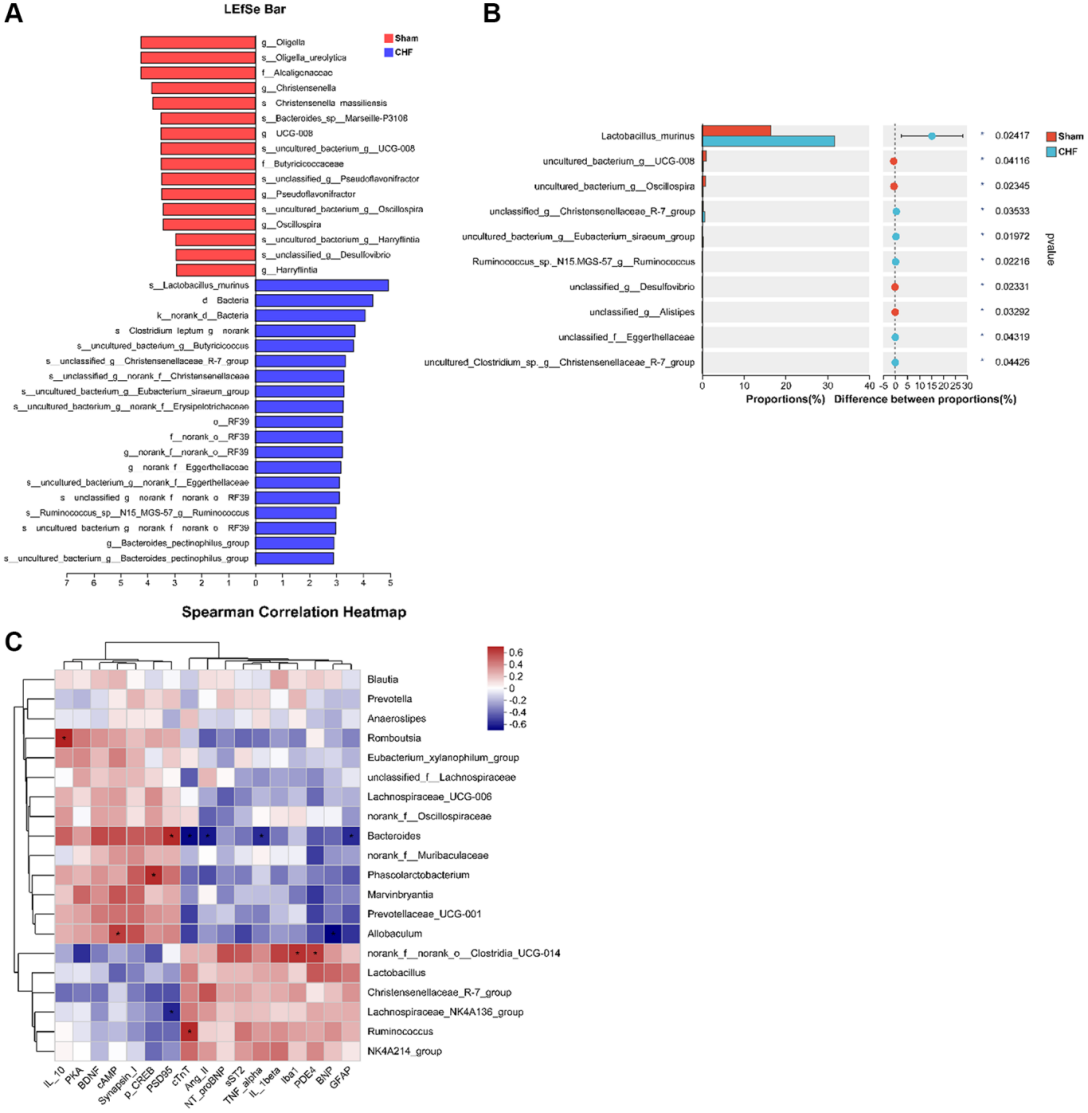
**Correlation between intestinal flora and inflammatory cytokines in the hippocampus.** (**A**) The linear discriminant analysis effect size (LEfSe) analysis at species level between Sham and CHF groups. (**B**) The top 10 species sorted by the relative abundance alterations between Sham and CHF groups. (**C**) Spearman analysis correlating differential flora and biochemical indicators of cardiac function, inflammatory cytokines, BDNF and key hippocampal proteins in the hippocampus. Data are presented as the Mean ± SEM; ^*^P < 0.05, and ^**^P < 0.01 vs. Sham; *n* = 6.

A LEfSe analysis identifies and interprets high-dimensional data to determine what features best explain species-to-species differences. We observed a difference between the two groups in the dominance of bacterial communities in our study comparing high-dimensional categories. Each group of bacterial genera is assigned a score based on its effects, with the linear discriminant analysis (LDA) score proportional to its significance. Intestinal flora community analysis with LEfSe revealed 36 species contributing to the relative abundances between Sham and CHF groups, with the Sham group contributing 16 species and the CHF group contributing 20 species, respectively (Figure 9A). There was variation in the gut microbiota caused primarily by *g_Oligella*, *s_Oligella_ureolytica*, *f_Alcaligenaceae*, *s_Lactobacillus_murinus*, *d_Bacteria, and k_norank_d_Bacteria* (Figure 9A). We analyzed the altered microbiota between the two groups. As sorted by relative abundance, the following 10 genera dominated the altered microbiota: *Lactobacillus murinus, uncultured bacterium g_UCG-008, uncultured bacterium g_Oscillospira*, unclassified g_Christensenellaceae_R7_group, uncultured bacterium g_Eubacterium sira-eum group (Figure 9B). We further explored the Spearman correlation between differential flora and cardiac indexes, inflammation, BDNF, and PDE4/cAMP/CREB/BDNF signaling. We analyzed the top 20 flora groups with altered abundance. For example, *Ruminococcus* is positively associated with cTnT. *Bacteroides* is negatively correlated with cTnT, Ang II, sST2 and GFAP, while it is positively correlated with PSD95 (Figure 9C).

## DISCUSSION

CHF is a complex clinical syndrome that is frequently accompanied by a range of complications, particularly cognitive impairment. Nevertheless, the precise mechanism underlying post-CHF cognitive impairment remains partially understood [44–46]. Our study demonstrates that CHF triggers an inflammatory state, disrupting hippocampus-dependent cognitive functions including spatial memory, learning behaviors, and locomotor activity. This impairment results from dendritic modifications and synaptic plasticity disorders due to neuroinflammation. Additionally, our research indicates a potential correlation between CHF-induced dysbiosis of intestinal flora subsequent to CHF and cardiocerebral indices. These findings offer new insights into the diagnosis and treatment of CHF and its concomitant cognitive dysfunction.

The predictors of CHF and cognitive impairment share similarities. Clinical evidence indicates that increased BNP/NT-proBNP may heighten the risk of cognitive impairment among individuals with CHF. Also, a low ejection fraction (EF < 30%) correlates with cognitive decline [47]. Recent studies have found a strong association between plasma sST2 level and neuronal damage and neurocognitive dysfunction [48, 49]. sST2 regulates β-amyloid pathology by controlling microglia activation and Aβ clearance [50]. It binds circulating IL-33, inhibiting intracellular IL-33 signaling [51]. Moreover, a correlation between plasma sST2 and TNF-α, Ang II levels has been observed [52, 53]. These markers could serve as promising biomarkers for post-CHF cognitive impairment.

A systemic inflammatory state significantly contributes to CHF’s pathological progression and complications. Increased inflammatory factors in CHF patients, including IL-1β, IL-6, TNF-α, C-reactive protein, immunoglobulin-like transcript 6 and myeloperoxidase, etc., can cross the blood-brain barrier, activating microglia and astrocytes, leading to neuroinflammation [54–56]. Numerous studies have demonstrated that an imbalance of IL-4, IL-6, IL-13, TNF-α, and IL-1β in the CNS contributes to neuropsychiatric disorders induced by MI [31, 32]. In the study, we detected elevated IL-1β, TNF-α, and, in particular, mildly elevated IL-10 in the hippocampus, further indicating persistent chronic inflammation.

Hippocampal lesions hinder the acquisition of new episodic memories. Previous research has established the hippocampal synapses’ role in memory, characterized by activity-dependent synaptic plasticity [57, 58]. Synaptic plasticity falls into two categories: structural and functional. The former pertains to adaptive modifications in synaptic ultrastructure, including synaptic quantity, density and dispersion, which ultimately determine the strength of synaptic connections [59]. Abnormal synaptic plasticity includes aberrant dendritic spine morphology, reduced synaptic density, synaptic loss, and irregular signal transduction. Dendrites’ length and pattern determine their synaptic input capacity. Our investigation suggests that CHF causes detrimental synaptic alterations in the hippocampus, including structural synaptic deficiency, diminished density and dendritic spines’ quantity. Dendritic spines, minute protrusions on the dendritic branches of excitatory neurons, are crucial to synaptic ultrastructure and form the postsynaptic structural basis for the majority of excitatory synapses [60].

Microglia, the immune cells in the brain, are indispensable for neuronal function and synaptic plasticity [61]. External stimuli polarize microglia towards a proinflammatory phenotype, activating inflammation, oxidative stress, and cytotoxicity, leading to neuronal death [62]. Astrocytes, the most abundant and diverse glial cells in the CNS, engage in the neurotransmitter metabolic processes (Glu, ATP, and GABA), and communication with neurons through neurotransmitters and neuromodulators, forming “tripartite synapses” [63, 64]. Proinflammatory microglia release various mediators, triggering reactive astrocyte transformation and neuroinflammatory cascades [65]. Microglia and neurons communicate through physical interactions and various receptors and signaling pathways. Co-culturing BV2 microglia with HT22 hippocampal neural cells offers a valuable model for investigating microglial inflammatory responses and neuronal interactions within the CNS. Our study offers a credible rationale for the effect of immune function on synaptic plasticity in CHF.

Notably, cAMP signaling plays a vital role in brain physiological mechanisms, involving neuronal activity, energy production, metabolic processes, and synaptic function. A decline in cAMP levels deactivates PKA, reducing phosphorylated CREB (p-CREB) level [66, 67]. The reduction in p-CREB levels, a nuclear transcription factor for learning, memory, and cognitive function, leads to decreased neurotrophic factors, particularly BDNF [68]. BDNF is essential for normal hippocampal function and supports hippocampal LTP [69, 70], maintaining dendritic spine density and facilitating memory-synaptic-specific protein synthesis [71]. Our study indicates that CHF reduces PKA activity, cAMP, BDNF, p-CREB levels, and synaptic-specific protein expression in the hippocampus. This result was further supported by *in vitro* co-culture of BV2 cells with HT22 cells. These results suggest that neuroinflammation induced by CHF or LPS adversely affects synaptic plasticity. PDE4, as a classical enzyme regulating cAMP hydrolysis, its inhibition mitigates memory impairments by reducing pro-inflammatory cytokines and oxidative stress, and enhancing neuronal activity in various conditions [72]. Our findings suggest that CHF increases hippocampal PDE4, and the dysregulated PDE4-dependent cAMP signaling might disrupt neuroplasticity in CHF. PDE4 might be a promising therapeutic target for post-CHF cognitive impairment. In recent years, some compounds including Rolipram, MK0952 and BPN14770, as PDE4 inhibitors, have been proven to alleviate neuroinflammation to enhance cognitive function [73]. However, transitioning these compounds from pre-clinical to clinical phases has been challenging, due to significant side effects [74]. Chinese herbal medicines and their active ingredients also provide new thoughts into drug development targeting PDE4. Over 50 natural PDE4 inhibitors have been identified, ranging from terpenoids, flavonoids and coumarins to novel polycyclic polypropylene acyl-phloroglucinol and diary fluorene derivatives [75]. RES003, a resveratrol derivative with an IC50 of 0.87 μM, can improve chronic stress-induced depression-like behaviors and other psychiatric disorders by inhibiting PDE4 and upregulating the p-CREB/BDNF signaling pathway [76]. Qishen Yiqi dropping pills, a Chinese herbal formula, reverse the upregulation of PDE4 to improve cognitive dysfunction related to heart failure and sleep deprivation [77]. The development of drugs targeting PDE4 could potentially address both cardiovascular and nervous systems simultaneously.

The nervous system and chemical substances facilitate communication between the intestine and brain [78]. It is posited that the inflammatory response and neurotrophic factors are intricately connected to the intestinal microbiota. The intestinal mucosa and blood-brain barriers allow immune and endocrine molecules to pass through, potentially affecting the functions of both the intestine and brain [79]. In addition, the intestinal microbiota can modify synaptic plasticity-associated proteins and receptors, including hippocampal N-methyl-D-aspartic acid (NMDA) receptors, serotonin receptors, and BDNF. Changes in the expression of these molecules may increase susceptibility to “risk behavior” and impair learning and memory [80]. Spearman analysis revealed that those intestinal flora associated with worsening cardiac indicators or inflammatory factors coincidentally are negatively associated with key synaptic proteins and cAMP pathway proteins. *Bacteroides*, a type of Gram-negative, non-spore-forming, anaerobic, and rod-shaped bacteria that constitute the resident flora in the human intestine, have been linked to CHF. A decrease in *Bacteroides* is associated with CHF, while its high level may improve cognitive and language development in infants, aligning with our findings [81, 82]. These findings imply that the intestinal flora may serve as a conduit for facilitating communication between the heart and brain.

Nevertheless, our research is ongoing, and further investigation is needed. Our study focused on the early stage of CHF, but further elucidation of cognitive impairment in the later stages of CHF is required. We concentrated on structural synaptic plasticity and future research will address functional synaptic plasticity, including LTP and LTD, in more detail. Although we have shown evidence of astrocyte and microglia activation, additional inquiry is necessary to fully understand the molecular mechanisms and other cellular modifications that occur in CHF. Identifying differences in intestinal flora between disease and health statuses has been challenging due to difficulties in precise measurement of individual flora composition. Significant advancements are pressing before this research can inform the prevention, diagnosis, and personalized treatment of CHF and cognitive impairment.

## CONCLUSION

**Figure 10:**
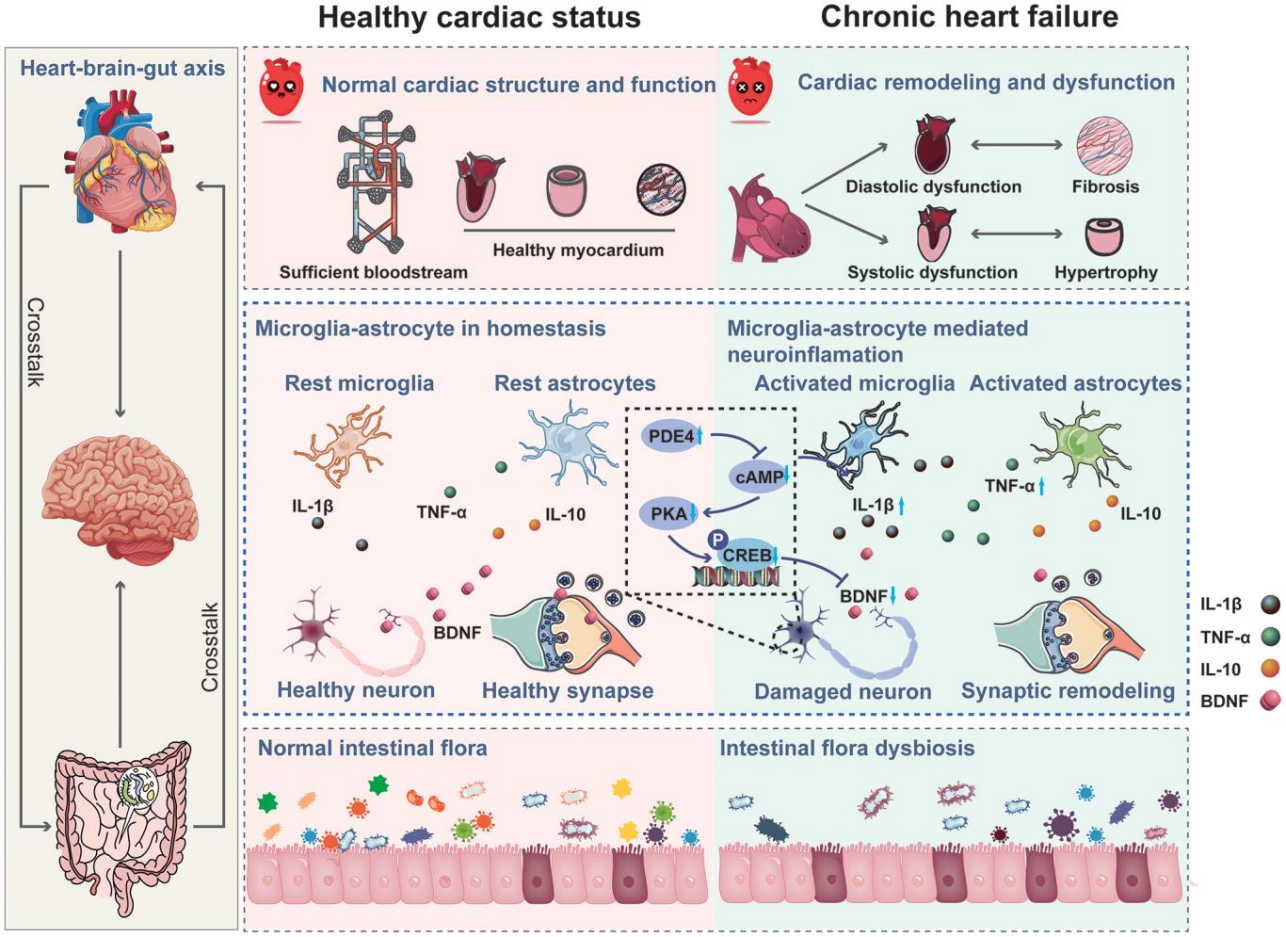
Mechanism of chronic heart failure induced cognitive impairment via impairing synaptic plasticity due to neuroinflammation associated with intestinal flora dysbiosis.

Collectively, our study confirmed that CHF impairs learning and memory in rats by promoting neuroinflammation in the hippocampus and causing intestinal dysbiosis. Neuroinflammation-induced alterations in structural synaptic plasticity may underlie hippocampus-dependent cognitive impairment following CHF, potentially implicating the PDE4-dependent cAMP signaling. Thus, inhibiting neuroinflammation, enhancing neuronal activity in the hippocampus, and improving intestinal flora could be effective therapeutic strategies for post-CHF cognitive impairment (Figure 10).
